# Many non‐native plant species are threatened in parts of their native range

**DOI:** 10.1111/nph.70193

**Published:** 2025-05-04

**Authors:** Ingmar R. Staude, Matthias Grenié, Chris D. Thomas, Ingolf Kühn, Alexander Zizka, Marina Golivets, Sophie E. H. Ledger, Laura Méndez

**Affiliations:** ^1^ Institute of Biology, Leipzig University 04103 Leipzig Germany; ^2^ German Centre for Integrative Biodiversity Research (iDiv) Halle‐Jena‐Leipzig 04103 Leipzig Germany; ^3^ Université Grenoble Alpes, University of Savoie Mont Blanc, CNRS, LECA Grenoble 38000 France; ^4^ Leverhulme Centre for Anthropocene Biodiversity, Department of Biology University of York York YO10 5DD UK; ^5^ Helmholtz Centre for Environmental Research – UFZ 06120 Halle Germany; ^6^ Institute of Biology/Geobotany and Botanical Garden, Martin Luther University Halle‐Wittenberg 06108 Halle Germany; ^7^ Philipp University of Marburg 35032 Marburg Germany; ^8^ Institute of Zoology, Zoological Society of London London NW1 4RY UK

**Keywords:** alien species management, biodiversity redistribution, biotic novelty, conservation paradox, extinction risk, range shifts

## Disclaimer

The New Phytologist Foundation remains neutral with regard to jurisdictional claims in maps and in any institutional affiliations.

Global change is reshaping plant biogeography, with ever more plant species becoming non‐native somewhere (Seebens *et al*., 2017). A key unresolved question is whether plants with non‐native ranges also thrive in their native ranges, as often hypothesized (Paudel *et al*., 2024), or if their extralimital success coincides with population declines at home. The potential ‘conservation paradox’ (Marchetti & Engstrom, 2016) – where species establish populations outside their native ranges while facing threats within – has been observed in various animal groups (e.g. mammals, birds, reptiles and amphibians; Gibson & Yong, 2017; Lundgren *et al*., 2024), but has never been assessed globally in plants. Assessing the prevalence of this pattern adds an important dimension to how we understand non‐native populations amid rapid biodiversity redistribution.

Here, we examine the global extent of naturalized species (plants with self‐sustaining non‐native populations) that simultaneously face threats in their native ranges (Supporting Information Methods S1). We collated subglobal Red Lists for vascular plants from 103 countries and combined them with the Global Naturalized Alien Flora (GloNAF) database (van Kleunen *et al*., 2019), which tracks naturalizations in 176 countries. Species names were harmonized using the World Checklist of Vascular Plants (Govaerts *et al*., 2021). While GloNAF offers near‐global data coverage, Red List data remain incomplete in parts of Africa and tropical Asia (Fig. S1), and assessment completeness varies across countries for both datasets (Methods S1). Nevertheless, these data offer the most comprehensive view on this topic to date.

**Fig. 1:**
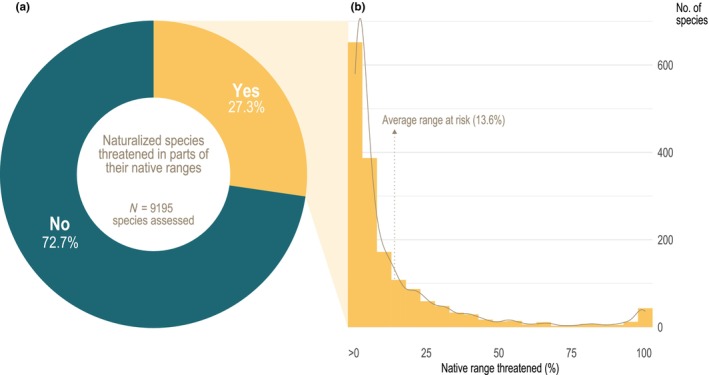
Threats to naturalized plant species within their native ranges. (a) The proportion of naturalized plant species threatened in part of their native range. (b) The distribution of the percentage of their native area of occupancy classified as threatened (native range threatened), based on Global Biodiversity Information Facility (GBIF) species occurrence records. The dotted arrow indicates the mean percentage (13.6%) of native range threatened. Histogram bin width: 5%.

Among the 9195 naturalized plant species world‐wide (excluding hybrids and apomictic genera), we found that 27.3% (*n* = 2513) are considered threatened in at least one country in their native range (Fig. 1a), based on national classifications standardized to International Union for Conservation of Nature (IUCN) global categories: Extinct, Critically Endangered, Endangered, or Vulnerable (Methods S1). Including Near Threatened (NT) species raises this to 31.1% (*n* = 2862). See Notes S1 for a discussion of potential under‐ or overestimation and underlying data considerations. This marked overlap between naturalization and subglobal threat highlights that range expansions and contractions often occur simultaneously – over one in four species with non‐native populations are threatened somewhere within their native range.

**Fig. 2:**
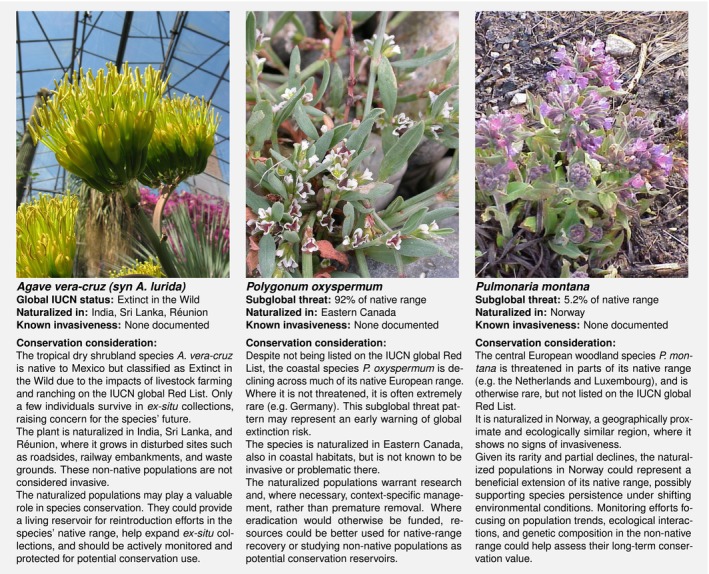
Three examples of the conservation paradox. Each species has non‐native populations but faces threats in its native range, reflecting a continuum from global to partial risk and highlighting the need for context‐specific conservation attention. Note that naturalization records reflect available data in GloNAF, and subglobal threat percentages are based on Supporting Information Methods S3. Photo credits: *Agave vera‐cruz* © Botanischer Garten TU Darmstadt, CC BY 2.0, via Wikimedia Commons. *Polygonum oxyspermum* © Olivier Pichard, CC BY‐SA 3.0, via Wikimedia Commons. *Pulmonaria montana* © Kurt Stüber, CC BY‐SA 3.0, via Wikipedia.

However, subglobal Red Lists do not capture species‐level extinction risk. We conducted an additional analysis using the IUCN global Red List that assesses threat status across species' full native ranges (Methods S2). This analysis shows that 2.1% (2.9% with NT species), or 1 in 50 naturalized species is *globally* threatened (seven even listed as Extinct in the Wild; Methods S2) – a more conservative estimate than the 1 in 4 found to be threatened in parts of their native range based on subglobal lists. Both perspectives are valuable. While the global Red List helps pinpoint species whose non‐native populations may warrant potentially urgent conservation attention, subglobal assessments expose earlier, spatially explicit contractions, offering a critical lens into how species ranges shift long before global status reflects it (Fig. 2).

To further assess the spatial extent of threat among the naturalized species with subglobal threats, we calculated their area of occupancy (AOO) within both threatened and non‐threatened parts of their native ranges. AOOs were calculated at a resolution of *c*. 6000 km^2^ at the equator, using occurrence records from the Global Biodiversity Information Facility (GBIF; Methods S3). We obtained AOO estimates for 1716 (68%) species with both naturalized and partially threatened native ranges. On average, these species faced threats across 13.6% of their native AOO, with half considered threatened in < 4.6% (Fig. 1b). This suggests naturalized species consistently threatened across their entire native ranges are relatively rare, echoing previous studies on extinction risk distribution (Channell & Lomolino, 2000; Holz *et al*., 2022).

**Fig. 3:**
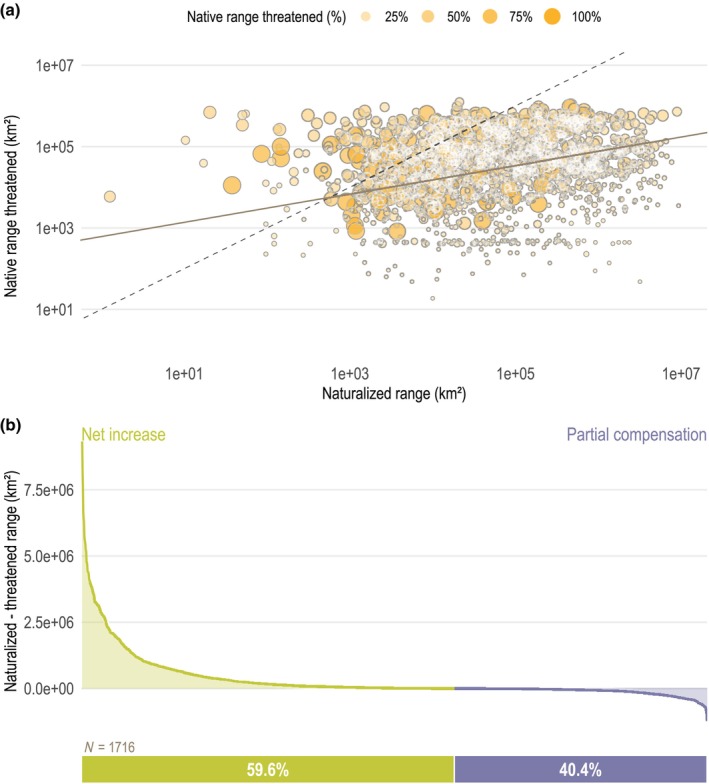
Naturalized ranges may partially compensate for threatened native areas. (a) Major axis regression comparing the sizes (measured as area of occupancy; AOO) of species' threatened native range vs naturalized range (solid line). Points are colored and sized according to the percentage of the native range classified as threatened. The dashed line represents the 1 : 1 ratio, where species have equal naturalized and threatened range sizes. Both axes are on a log_10_ scale. (b) Distribution of differences between naturalized and threatened ranges. Of 1716 naturalized species analyzed (Supporting Information Methods S3), 40.4% (*n* = 693) have smaller naturalized ranges than threatened ones (partial compensation, blue), while 59.6% (*n* = 1023) have larger naturalized ranges (net increase, green).

We further evaluated the balance between species' naturalized and threatened AOOs for the subset of 1716 naturalized species that are threatened in part of their native range and for which occurrence records are available. Major axis regression on log_10_‐transformed values revealed a positive relationship with a slope significantly below 1 (slope = 0.35; 95% CI = 0.25–0.46; Fig. 3a), suggesting that while small gains in naturalized ranges can sometimes be offset by larger losses in native ranges, naturalized AOOs generally exceed threatened AOOs. A paired Wilcoxon test confirmed this, showing that the median naturalized AOO (60 593 km^2^) was significantly larger than the median threatened AOO (37 066 km^2^; *P* < 0.001, Fig. S2). Thus, on average, these species occupy more area in their naturalized than in their threatened native range, and therefore do not experience net range losses.

These patterns form part of a broader continuum of species with expanding, contracting, or mixed range dynamics. Across our full dataset (Methods S1), 22 789 species are threatened in at least part of their native range but lack known non‐native populations (net loss), while 6682 species are not threatened in their native range but are naturalized elsewhere (net increase). The 2513 species that are both naturalized somewhere and threatened in part of their native range fall in between. Among those with occurrence data, 40.4% have smaller naturalized AOOs than threatened native AOOs (partial compensation), while 59.6% have larger naturalized AOOs (net increase; Fig. 3b). This variation highlights that while naturalized populations can offset range losses for some species, they do not do so consistently.

We further investigated whether specific plant families or countries disproportionately contribute to the pool of naturalized species threatened somewhere in their native ranges. Phylogenetic relationship did not explain which plant families contain these threatened and naturalized species, as assessed by using three different phylogenetic metrics (Methods S4). Instead, they are broadly distributed across the phylogenetic tree of seed plants, occurring in 74% of all families that include species with non‐native ranges (Fig. S3). Across countries, an average of 29% of the naturalized flora consists of species with native range contractions. Most European countries exceed this average, with seven countries having over half of their naturalized flora classified as threatened in parts of their native ranges (Fig. S4). Such regions may increasingly contribute to navigating the conservation paradox.

Our findings highlight an aspect that is rarely considered in broader discussions surrounding non‐native species: one in four not only thrive abroad but are also threatened in parts of their native ranges. This conservation paradox in plants mirrors patterns seen in mammals, where 22% of species with introduced ranges are also threatened in their native ranges (Lundgren *et al*., 2024). As ever more plant species are predicted to be at risk (Bachman *et al*., 2024) and as subglobal Red Lists and naturalization data improve, the overlap between non‐native and threatened species and the prevalence of such ‘paradoxical’ species are poised to increase. Yet, native and non‐native populations are not always ecologically equivalent, as they interact with different communities. This begs the question: should non‐native populations of these species be considered in conservation efforts, and if so, how?

Addressing this question requires placing it within the broader context of global change. The Earth is experiencing rapidly increasing biotic novelty, as species shift ranges in response to climate change and continue to move individually and asynchronously into no‐analog assemblages, as they have during past climatic shifts (Williams & Jackson, 2007; Ordonez *et al*., 2024). In such settings, the concept of nativeness becomes less clear‐cut, and rigid adherence to it may obscure opportunities to support ecosystems. Some non‐native populations may offer functional traits that help buffer ecosystem processes against novel future environments, particularly where native species lack such traits (Schlaepfer *et al*., 2011). Conservation decisions involving non‐native populations of species declining elsewhere may therefore need to account for this ecological novelty while still applying caution (Kerr *et al*., 2025).

Above all, evaluations of species with non‐native ranges need to be made at the population level, not generalized across all non‐native populations. While naturalizations in biogeographically similar regions may pose lower ecological risks, introductions into eco‐evolutionary novel environments warrant caution (Essl *et al*., 2019). In such ecosystems, non‐natives may evolve distinct genotypes or become invasive, disrupting native communities (Saul & Jeschke, 2015). This creates difficult questions: Should a non‐native population be protected if doing so risks harm to a native ecosystem? And if threatened species thrive elsewhere, might conservation agencies deprioritize local efforts, inadvertently shifting responsibilities? How can conservationists, already on tight budgets, be motivated and funded to protect non‐native populations, some of which may be ecologically harmful?

Conservation translocations already move species beyond historical ranges due to climate change, offering a model for integrating non‐native populations into conservation planning (Gaywood *et al*., 2022). Yet, limited data on whether non‐native populations integrate functionally or disrupt ecosystems (e.g. via trophic interactions or competition) complicate decision‐making. New interaction databases (e.g. on plant‐microherbivore interactions; https://bladmineerders.nl/) may offer insights into ecological fit. Where fit is likely, non‐native populations could contribute *in situ* to biodiversity and ecosystem function. If persistence outside the native range is crucial for global conservation but poses ecological risks, *ex situ* conservation may be preferable. A global conservation network could coordinate regions where species decline in their native range with those where they thrive as non‐natives, integrating local and global conservation priorities.

Here, we show that many plants with non‐native ranges experience native range contractions. While we do not argue that non‐native populations should generally be seen as opportunities rather than threats to conservation, an overly rigid view of what is considered native – and thus worthy of conservation – risks overlooking the dynamic nature of species ranges, the subjectivity of ecosystem membership, the historical role of long‐distance dispersal in shaping biodiversity, and the uncertainty around what is even native to begin with (Pereyra, 2020). Our analysis does not dismiss the importance of assessing species' origins, traits, impacts, and ecological histories. Rather, we call for a more nuanced view, one that acknowledges that non‐native populations may, in some cases, offer conservation benefits alongside risks. When assessing their ecological impact, a complementary question to ask is: might there also be value for conservation?

## Competing interests

None declared.

## Author contributions

IRS conceived the project, led the analysis, and coordinated the manuscript writing. LM led data synthesis efforts with support from Matthias Grenié. IRS, Matthias Grenié, CDT, IK, AZ, Marina Golivets, SEHL and LM contributed to writing the manuscript.

## Supporting information


**Fig. S1** Geographic distribution of data availability for the study.
**Fig. S2** Bland–Altman plot comparing species' naturalized and threatened range size.
**Fig. S3** Phylogenetic distribution of plant species that are naturalized and threatened.
**Fig. S4** Percentage of naturalized plant species per country that are threatened elsewhere.
**Methods S1** Data compilation.
**Methods S2** Contrasting global and national assessments.
**Methods S3** The spatial extent of threat in naturalized plant species with subglobal threats.
**Methods S4** Phylogenetic patterns in naturalized species threatened at home.
**Notes S1** Considerations for estimating the prevalence of the conservation paradox.
Please note: Wiley is not responsible for the content or functionality of any Supporting Information supplied by the authors. Any queries (other than missing material) should be directed to the *New Phytologist* Central Office.

## Data Availability

All data and code to reproduce the findings of this study are available on GitHub at: https://github.com/istaude/threatened‐nonnatives.
