# Supplementary material for: Many non‐native plant species are threatened in parts of their native range
Source: New Phytol. 2025 May 4;247(4):1579–83. doi: 10.1111/nph.70193 (PMC12267924; doi:10.1111/nph.70193)
Supplement: Supplementary file 1 — Fig. S1 Geographic distribution of data availability for the study. Fig. S2 Bland–Altman plot comparing species' naturalized and threatened range size. Fig. S3 Phylogenetic distribution of plant species that are naturalized and threatened. Fig. S4 Percentage of naturalized plant species per country that are threatened elsewhere. Methods S1 Data compilation. Methods S2 Contrasting global and national assessments. Methods S3 The spatial extent of threat in naturalized plant species with subglobal threats. Methods S4 Phylogenetic patterns in naturalized species threatened at home. Notes S1 Considerations for estimating the prevalence of the conservation paradox. Please note: Wiley is not responsible for the content or functionality of any Supporting Information supplied by the authors. Any queries (other than missing material) should be directed to the New Phytologist Central Office. [file NPH-247-1579-s001.pdf]

New Phytologist

***New Phytologist* Supporting Information**

Article title: Many non-native plant species are threatened in parts of their native range
Authors: Ingmar R. Staude, Matthias Grenié, Chris D. Thomas, Ingolf Kühn, Alexander Zizka, Marina Golivets, Sophie E. H. Ledger, Laura Méndez
Article acceptance date: 16 April 2025

Contents:

Methods
S1: **Data compilation.**
S2: **Contrasting global and national assessments.**
S3: **The spatial extent of threat in naturalized plant species with subglobal threats.**
S4: **Phylogenetic patterns in naturalized species threatened at home.**

Notes
S1: **Considerations for estimating the prevalence of the conservation paradox.**

Supplementary Figures
Fig. S1: **Geographic distribution of data availability for the study.**
Fig. S2: **Bland–Altman plot comparing species' naturalized and threatened range size.**
Fig. S3: **Phylogenetic distribution of plant species that are naturalized and threatened.**
Fig. S4: **Percentage of naturalized plant species per country that are threatened elsewhere.**

Methods

Methods S1: **Data compilation and integration**

Red List data synthesis. We integrated subglobal Red Lists (RLs) from various sources, each representing prior efforts to compile RLs at different geographic scales. European data were obtained from Holz *et al.*, 2022, Russian data were obtained from Xue *et al.*, 2023, and North American data were retrieved from NatureServe (https://www.natureserve.org). Global data were accessed via the archived National Red List website (https://www.archive.nationalredlist.org; ZSL and IUCN National Red List Working Group, 2022), with additional global data (Schippmann, 2020) kindly provided by Uwe Schippmann. Compiled data were aggregated, and region and country names were standardized to address inconsistencies in nomenclature across different RLs. In total, we compiled RL data for 103 countries (Fig. S1).

We harmonized species names across RLs following best practices (Grenié *et al.*, 2023). First, we standardized taxon name formats using the *name_parse()* function from the rgbif R package (Chamberlain *et al.*, 2022), preserving author names when available. To improve name matching, we assigned taxonomic groups using *name_backbone_checklist()*, which references the Global Biodiversity Information Facility (GBIF) backbone taxonomy to identify higher taxonomic ranks. This process identified several phyla (e.g., Arthropoda, Ascomycota), which we excluded, retaining only Tracheophyta (vascular plants). We also kept species absent from the GBIF database, attributing their absence to potential inaccuracies or misspellings. Finally, we harmonized taxonomies using the World Checklist of Vascular Plants (WCVP) (Govaerts *et al.*, 2021), employing the *wcvp_match_names()* function from the rWCVP and rWCVPdata packages (Brown *et al.*, 2023) with exact matching and author names where available.

Country-specific RL categories were standardized to global International Union for Conservation of Nature (IUCN) categories for consistency (e.g., see Holz *et al.*, 2022 for a crosswalk used in several European countries, which we followed). Species were considered threatened if they fell under any of the IUCN categories of Extinct (EX), Critically Endangered (CR), Endangered (EN) or Vulnerable (VU) in at least one country/region. In total, there were 51,426 threatened species-country/region combinations: 4,161 (8.1%) were classified as Extinct, 5,770 (11.2%) as Critically Endangered, 19,197 (37.3%) as Endangered, 16,195 (31.5%) as Vulnerable, and 6,103 (11.9%) as "threatened" without IUCN category (i.e., these instances were difficult to match to an IUCN category, e.g., Nationally Critical, Declining, etc., but were indicative of a "threatened" status). All RL categories were subsequently consolidated into a single "threatened" category. In a supplementary analysis (see below), we also included species classified as near threatened, totaling 12,532 species-country/region combinations. All non-threatened species were excluded.

We excluded hybrids (161 spp.), subspecies and varieties (3,307 spp.) from our data. Additionally, we excluded species from apomictic genera, as these can massively inflate extinction rates [e.g., Hieracium in Sweden (Holz *et al.*, 2022)]. We used data from Hojsgaard *et al.*, 2014 and R code from Schrader *et al.*, 2024[i] to flag and remove species from apomictic genera (5,365 spp.). In total, 25,428 unique species were classified as threatened (and 3,235 as exclusively near threatened) in at least one of 103

---

i https://springernature.figshare.com/articles/dataset/Supplementary_data_to_Islands_are_key_to_protect_the_world_s_plant_endemism/24448108

countries. Removing hybrids, subspecies, varieties and species from apomictic genera led to a 22% reduction of distinct, red-listed species in our data (from 36,710 to 28,633).

Red List data description. There were data gaps for Africa and tropical Asia in the current sources. While Red Lists for these regions may exist, they are either not available online, not yet processed into databases due to limited capacity, or excluded due to biases around non-English sources. Ethiopia and Eritrea shared a combined RL but are counted here as lists for two countries. Some countries did not have data on all threatened species; for instance, the lists compiled by Schippmann, 2020 include only extinct (or possibly extinct) species. In cases where this was the only available data (e.g., Argentina), the country's RL was represented solely by extinct species. Additionally, some national RLs were particularly incomplete, such as Thailand's, which included only seven species. The general variation in data quality stemmed from two main sources: 1) differences in data collation, as the sources we used may not have aggregated all available data, and 2) variation in the completeness of national or subnational assessments. These distinctions are often difficult to separate. As a result, the completeness of RLs varied greatly across regions.

RL publication years ranged from 1985 (Portugal, but a newer list was also available) to 2022 (Austria), with a median publication year of 2010 (Methods S1 Figure). For 42 countries, we had multiple lists spanning different years (but note that not all lists had a publication year, as not all the sources mentioned above included this information). In 31 of these cases, the lists did not appear to be comprehensive RLs, as multiple publication years fell within a five-year period and listed only a few species. Instead, these lists reflect data aggregated by https://www.nationalredlist.org from diverse sources, including assessments by governmental agencies, where the objective and focus might have been on fewer or specific target species. For 56 countries, we had lists from multiple sources. We aggregated data across both years and sources. Combining data from multiple years may lead to an inflation in the count of threatened species, as downlisted species (i.e., previously listed as threatened becoming listed as non-threatened) could still be counted. We excluded such species from the respective RLs. In cases of conflicting information across sources, we conservatively selected the assessment with the least severe RL category.

For some countries, we were able to retrieve subnational assessments. Where national assessments were available (e.g., Germany), we excluded subnational RLs to avoid inflating the counts of red-listed species. However, in countries such as Belgium, Canada, China, India, Mauritius, Russia, the United Kingdom, and the United States of America (USA), only regional RLs were available, which we aggregated at the ISO3 level. In many cases, these regional RLs did not cover the entire country (e.g., data was available only for Southern China). Despite their incompleteness at the national level, these lists provided the only data available and were included in our analysis. For large countries like Russia, the USA and Canada, the subnational RLs were relatively comprehensive, covering 72 of 85 federal subjects (ISO2 level) in Russia (85%), all 50 states and the District of Columbia (100%) in the USA, resulting in 51 lists, and all 13 provinces and territories in Canada (100%). We maintained a separate dataset of these subnational (ISO2 level) RLs for later analysis [e.g., to avoid the use of ISO3-aggregated data to prevent inflated estimates of threatened ranges (see below)].

Naturalization data. For species naturalizations, we used data from the Global Naturalized Alien Flora (GloNAF) database (van Kleunen *et al.*, 2019), which compiles naturalization events worldwide. As with our RL data, the completeness of the naturalization records varies by region, with a strong bias toward

more comprehensive coverage in Europe and North America. As with our RL data preparation, we used the rWCVP R package (Brown *et al.*, 2023) to harmonize species names, prioritizing accepted names when multiple matches were available. We included only species classified as "naturalized" [excluding those classified as "alien" (n = 766)]. We excluded hybrids (n = 361), subspecies and varieties (647 spp.), and species from apomictic genera (n = 2,111) to ensure consistency with our RL data, where these species were also excluded. This resulted in a final dataset of 9,195 species naturalized in at least one of 176 countries. Note that GloNAF occasionally records naturalization at varying spatial scales. That means species may be recorded as naturalized at a regional rather than a national level, which can inflate the count of non-native species when subnational lists are aggregated to the ISO3 level. As a result, aggregating GloNAF data at the ISO3 level may not perfectly align with national reports for countries (when GloNAF only provides subnational assessments, for example, for Germany). Still, this aggregation was necessary to match our RL data.

Combining Red List and naturalization data. To integrate the RL and naturalization datasets, both containing the columns for species, country (ISO3), and status (threatened or naturalized), we combined their rows. There were 624 species classified, contradictorily, as both non-native and threatened within the same ISO3 region, which could arise from two possible scenarios. For instance, *Abies alba* is correctly classified as threatened in southern Germany but non-native in the north—a distinction lost when aggregating data at the national scale. Alternatively, some non-native species may have been included in national RL assessments. The second scenario is problematic for our analysis, which aimed to identify how many non-native species are threatened in their native range.

To resolve which species fell under the latter scenario, we consulted a third dataset: WCVP (https://powo.science.kew.org/). Available via the *wcvp_distribution()* function in the rWCVP package (Brown *et al.*, 2023), we obtained species' native range shapefiles (i.e., combined polygons of all botanical countries where a species is native) and tested for overlap between a species' native range and the ISO3 country where it was listed as both threatened and non-native, utilizing the sf package's *st_intersection()* function(Pebesma, 2018). We retained species with overlapping native ranges and discarded the country-threatened species combinations without native range overlap. A secondary finding from this analysis is the identification of 500 species globally that are genuinely classified as both non-native and threatened within the same country.

That said, our approach may have overlooked a third possible scenario: a species might be classified as threatened but not recorded as a naturalized species for that country in GloNAF, despite being non-native there. Excluding such cases is challenging, as WCVP's native range data is not complete, and consistently cropping RL data risks omitting valid cases where a species is both native and threatened. To address this, we reviewed parts of our dataset (focusing on countries that list non-natives in their RLs; see above) and identified instances in an older Swiss RL (from 2002) with two species—*Scilla lucilae* (syn. *Chionodoxa luciliae*) and *S. forbesii* (syn. *C. forbesii*)—that were red-listed in Switzerland but native only to Turkey, without appearing as non-native in GloNAF. Despite these efforts, we caution that a few such cases may remain unaccounted for.

In total, we therefore count 25,302 (25,428 – 126 species red-listed but non-native in the listing country) species that are genuinely classified as threatened in at least part of their native ranges. Since our focus was on quantifying the global magnitude of naturalized species that are part-of-native-range-threatened, we excluded species that were exclusively threatened (or near threatened) and had not

naturalized anywhere (n = 22,789). We thus retained only exclusively non-native species and those that were both threatened (or near threatened) and non-native. Our final dataset comprised 48,835 species-country combinations, detailing countries where species are naturalized and threatened, including data for 2,513 threatened and 9,195 non-native species (when near threatened species were included as threatened species there were 50,750 species-country combinations for 2,862 threatened and 9,195 non-native species).

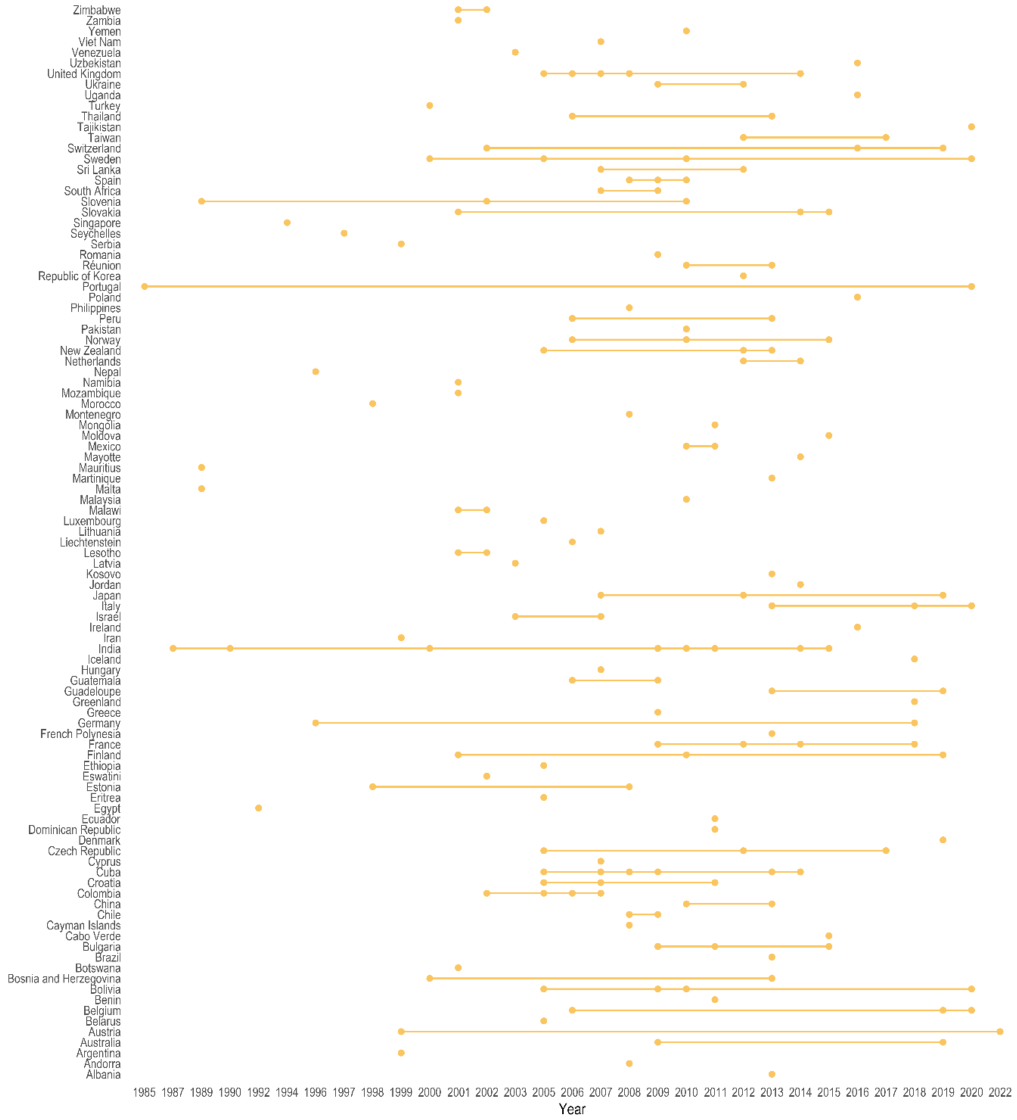

Methods S1 Figure: **Publication years of Red Lists.** Publication years of national Red Lists for vascular plants by country (y-axis). Each dot represents a publication year, with lines connecting multiple publication years within the same country. Red Lists published more frequently than every five years are often assessments by governmental agencies, where the objective and focus is on fewer or specific target species. Note: Only national Red Lists are included; subnational lists for Russia, the USA, and Canada are excluded.

Methods S2: **Contrasting global and national assessments.**

To provide a global contrast to national assessments, we used the IUCN global RL (IUCN, 2025). While still incomplete for plants, with only approximately 17% of global plant biodiversity assessed, it remains the most comprehensive source of global risk status for plant species. Following the same methodological steps as for (sub-)national RLs (Methods S1), we harmonized species names using the rWCVP R package (Brown *et al.*, 2023), excluded hybrids (n = 66), subspecies and varieties (n = 420), and species from apomictic genera (n = 9,072). Species were considered threatened if classified under any of the IUCN categories: Extinct (EX), Critically Endangered (CR), Endangered (EN), or Vulnerable (VU). In total, the dataset included 24,523 threatened species, distributed as follows: 159 (0.6%) Extinct, 5,257 (21.4%) Critically Endangered, 10,297 (42%) Endangered, and 8,810 (35.9%) Vulnerable. Additionally, 3,169 species were classified as Near Threatened (NT). We integrated this global RL dataset with the naturalization dataset (Methods S1) to identify species that are both globally threatened and naturalized beyond their historical range. We found that 2.1% of the global naturalized flora is globally threatened (n = 190 out of 9,195 species), with the majority classified as Vulnerable (83 species), Endangered (61 species), or Critically Endangered (38 species). Additionally, 1 species is classified as Extinct and 7 as Extinct in the Wild. Including Near Threatened species raises this figure to 2.9% (77 additional species).

Methods S3: **The spatial extent of threat in naturalized plant species with subglobal threats**

To calculate the area of a species' range that is threatened, the fraction of the native range at risk, and the area gained from naturalizations, we used species point occurrence records from GBIF. For each naturalized, part-of-native-range-threatened species, we obtained a GBIF "usageKey" using the *name_backbone()* function from the rgbif package (Chamberlain *et al.*, 2022), which interfaces with GBIF's taxonomic backbone. These keys were then used with *mvt_fetch()* to retrieve map vector tiles for each species. We parametrized the *mvt_fetch()* function as follows: 1) Only records with the basis of record as "human observation", "machine observation" and "observation" were included (i.e., fossil specimens, living specimens from zoos and botanical gardens, and preserved specimens from museums and herbaria were excluded); 2) Records flagged with coordinate issues were excluded; and 3) A square size of 8 (corresponding to a mapping resolution of approximately 6,000 km^2 at the equator, or 2,500 km^2 in Central Europe) was used for aggregation of occurrence records in Web Mercator (EPSG:3857) projection (see rdocumentation[ii] for details).

Species tiles were cropped and classified into three range categories: native and threatened, native and not-threatened, and non-native. For the threatened range, we used the dataset which retained both ISO-3 and ISO-2 codes (see above). This dataset conserved subnational assessments (with ISO-2 codes) for the large countries Canada, Russia, and the USA, providing better resolution that helps avoid overestimating the threatened area. We used these data in combination with the *ne_countries()* and *ne_states()* functions from the rnaturalearth package (Massicotte *et al.*, 2023), which provided the shapefiles for these ISO-3 and ISO-2 regions. To obtain the tiles where a species is threatened, we first intersected the species' threatened range with GBIF data. Second, we identified each species' native range using the *wcvp_distribution()* function from the rWCVP package (Brown *et al.*, 2023). This function generated a shapefile of botanical countries where the species is native, excluding introduced and extinct occurrence types. To ensure the native range always included all regions where the species is threatened, we combined the threatened ISO-3 and ISO-2 regions with the botanical countries of the native range for each species and intersected them with the GBIF data to obtain the species' native range. Finally, we used shapefiles from the GloNAF dataset (https://idata.idiv.de/DDM/Data/ShowData/257) to identify regions where species are non-native. These shapefiles include an OBJIDsic identifier that links to the corresponding GloNAF species region IDs. The corresponding shapefiles can be viewed here: https://sebastian-ch.github.io/glonafAtlas/continentView/index.html. Any tiles that did not fall within a species' native, threatened, or non-native range were cropped.

Since we wanted to quantify the range dynamics of naturalized, part-of-native-range-threatened species, we required each species to have at least one tile in each of the three range categories. Of the 2,513 species, 27 had no occurrence records in GBIF, 6 had no records in their native range, 383 had no records in their threatened range, and 515 had no records in their non-native range, leaving 1,716 species (68%) for analysis. We used the sf package (Pebesma, 2018), employing the *st_union()* and *st_area()* functions to calculate the area of these ranges. Certainly, these AOO estimates have limitations; for example, national red-listing does not necessarily indicate that a species is threatened across its entire range within a country. Additionally, distribution data are often incomplete and

[ii] https://www.rdocumentation.org/packages/rgbif/versions/3.8.0/topics/mvt_fetch

influenced by sampling gaps, biases, or, in our case, recent extirpations within threatened ranges. Nonetheless, these estimates offer a rough measure of the areas at risk and the areas gained.

New Phytologist

Methods S4: **Phylogenetic patterns in naturalized species threatened at home**

To assess the phylogenetic signal of the percentage of non-native species that are threatened at home at the family level, we used a phylogenetic tree with family-level resolution, including 371 seed plant families (Li *et al.*, 2021). We calculated three metrics of phylogenetic signal: Pagel's λ, Blomberg's K and the D-statistic. Pagel's λ and Blomberg's K were calculated using the *phylosig()* function from the phytools R package (Revell, 2012), while the D-statistic was computed using the *phylo.d()* function from the caper package (Orme *et al.*, 2013). Pagel's λ was 0.37 (logL = -19, P = 0.211), indicating no significant phylogenetic signal. Blomberg's K was 0.297 (P = 0.084, 1000 randomizations), suggesting weak but non-significant phylogenetic signal. The D-statistic tests the binary version of the percentage of species with non-native areas that are also considered threatened in their native ranges (where percentages > 0, were 1 and 0 = 0) was 1.17. The probability of this D resulting from random phylogenetic structure was 0.87, and from Brownian motion was 0. All tests provided support that the incidence and percentage of naturalized, part-of-native-range-threatened species does not exhibit a significant phylogenetic signal. Thus, phylogeny is not predictive of which plant families have non-natives that are threatened at home.

Notes

Notes S1: **Considerations for estimating the prevalence of the conservation paradox**

Our estimate may be conservative, as accessible data on naturalized species are more geographically complete than subglobal Red List data (Fig. S1). Limiting our analysis to countries with national assessments for both non-native and threatened species yielded an estimate of 27.1% (very similar to the full data set estimate of 27.3%). Conversely, it may be inflated. Large native range size increases the number of assessment opportunities, and species with non-native populations may also have large native ranges. Whilst we do not consider this to be a pure sampling effect because species with larger ranges are also exposed to more varied pressures (e.g., habitat loss, harvesting) and thus genuine, spatially explicit threats, we tested this idea (Notes S1 Methods). The world's most widespread plant species had zero probability of being considered threatened on a national Red List (Notes S1 Figure), indicating that a large native range size alone does not consistently predict subglobal threatened status. Another consideration arises for range-edge species, which may be (falsely) considered threatened on national Red Lists due to their restricted distribution within geopolitical borders. Although IUCN guidelines require that such assessments consider conspecific populations outside the region (IUCN, 2012), it remains unclear how consistently this is applied in practice. Together, there are several complexities and challenges in assessing genuine threat status amidst dynamic ranges, varying national listing criteria, and spatial factors such as species range size, position, and country boundaries—issues that also apply to non-native classifications (Essl *et al.*, 2018). Hence, uncertainties remain regarding the true proportion of naturalized species threatened elsewhere. Yet, the data that do exist show that over one in four naturalized plant species assessed for conservation status is classified as threatened within at least part of their native ranges.

Notes S1 Methods: **Assessing the influence of range size on red-listing probability**

For this analysis, we used data from WCVP (Brown *et al.*, 2023) to obtain a global list of all plant species. We again included only accepted species, excluded hybrids, subspecies, varieties, and apomictic genera, yielding a global pool of 316,982 plant species. WCVP also details the botanical countries where each species is native. Botanical countries, unlike political ones, are delineated to be more comparable in size. In this analysis, the number of botanical countries represents the range size of each species. We chose this metric as it was straightforward to calculate for the entire global flora. We combined this with our RL synthesis of 25,428 distinct threatened species, creating a binary indicator for whether a species was threatened or not.

To explore the relationship between range size and likelihood of being threatened, we plotted range size against this binary indicator with a LOESS regression, which revealed a hump-shaped trend (Notes S1 Figure a). Given the large sample size (n>300,000), even small effects would be statistically significant. Therefore, we binned the range sizes (1–360 botanical countries) into 50 equal-sized intervals and calculated the median range size and the proportion of species threatened within each bin. This approach produced 50 data points for median range size and probability of being threatened, which we then modelled using a generalized additive model (GAM; specified with a Gaussian distribution and identity link function) with a smooth term for range size (k = 10), using the mgcv package (Wood & Wood, 2015).

This analysis revealed a significant non-linear relationship between species range size and the probability of being threatened (edf = 6.37, F=39.8, p<0.001). The model explained 87.8% of the deviance, with an adjusted R^2 of 0.859. Species with intermediate range sizes had the highest probability of being threatened in at least one country, while species with very small or very large ranges exhibited lower probabilities. This pattern suggests that large-ranged species are not consistently threatened, thereby challenging the null expectation (Notes S1 Figure b).

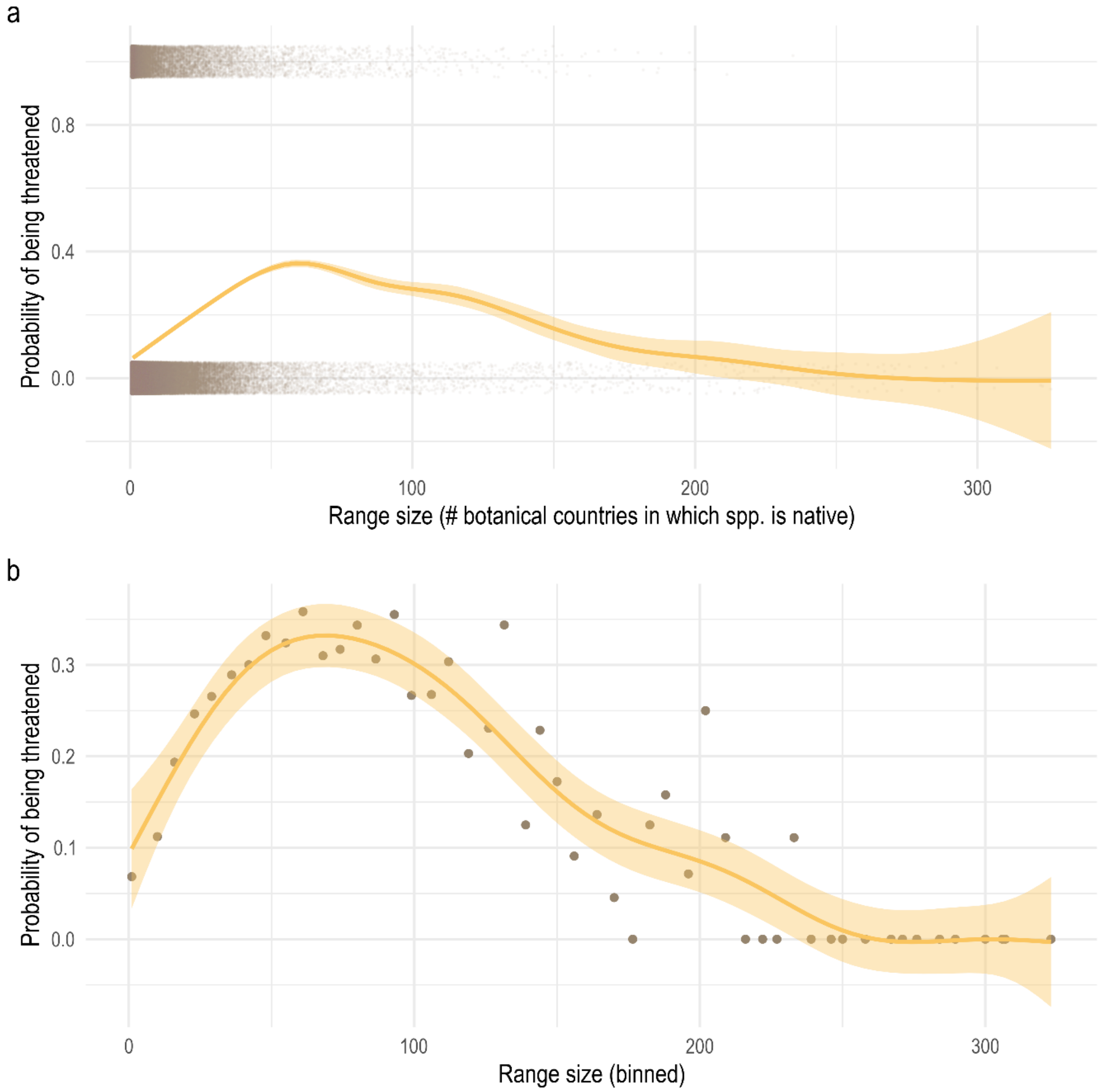

Notes S1 Figure: **Relationship between species range size and the likelihood of being listed as threatened on a subglobal Red List. a,** Raw data plot for 316,982 plant species showing the binary outcome of whether a species is threatened in at least one country (yes = 1, no = 0) against the number of botanical countries where a species is native (range size). The yellow line indicates an exploratory local regression to illustrate trends. **b,** Generalized Additive Model fitted to binned data, where range sizes were grouped into 50 equal intervals. For each bin, the median range size and the fraction of species threatened were calculated. Shaded ribbons in **a** and **b** indicate 95% confidence intervals around the predicted line.

New Phytologist

Supplementary Figures and Legends

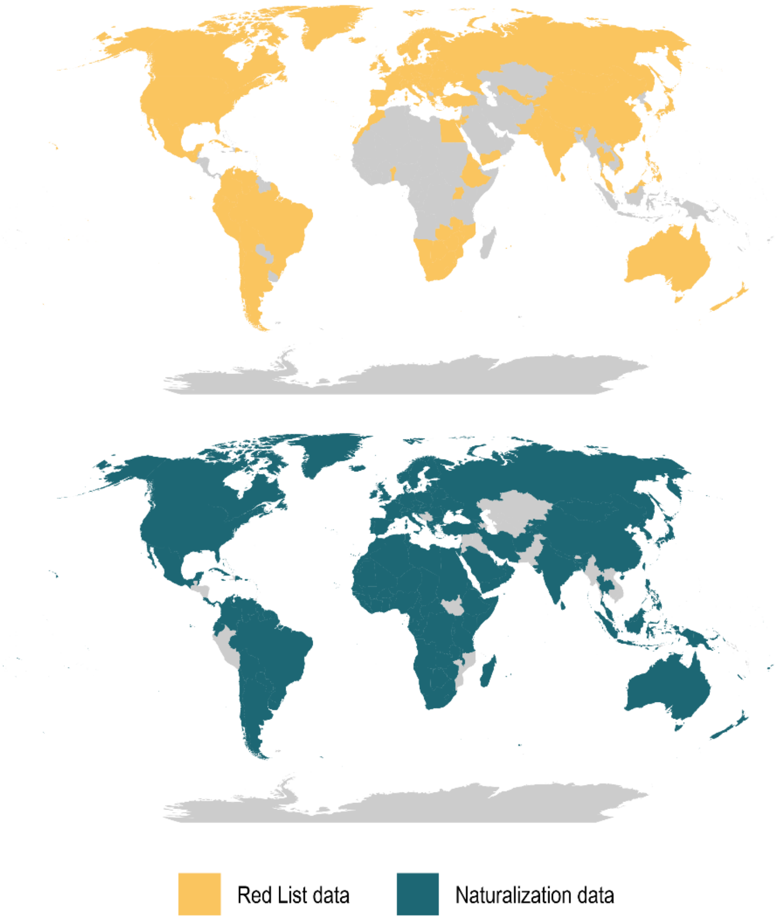

Fig. S1: **Geographic distribution of data availability for the study.** The top map (in yellow) shows the 103 countries for which Red List data were available. The bottom map (in green) highlights the 176 countries included in the GloNAF dataset for naturalized non-native species (after excluding hybrids, subspecies, varieties and apomictic genera; see Methods S1). Gray areas indicate countries where data were not available in the respective datasets.

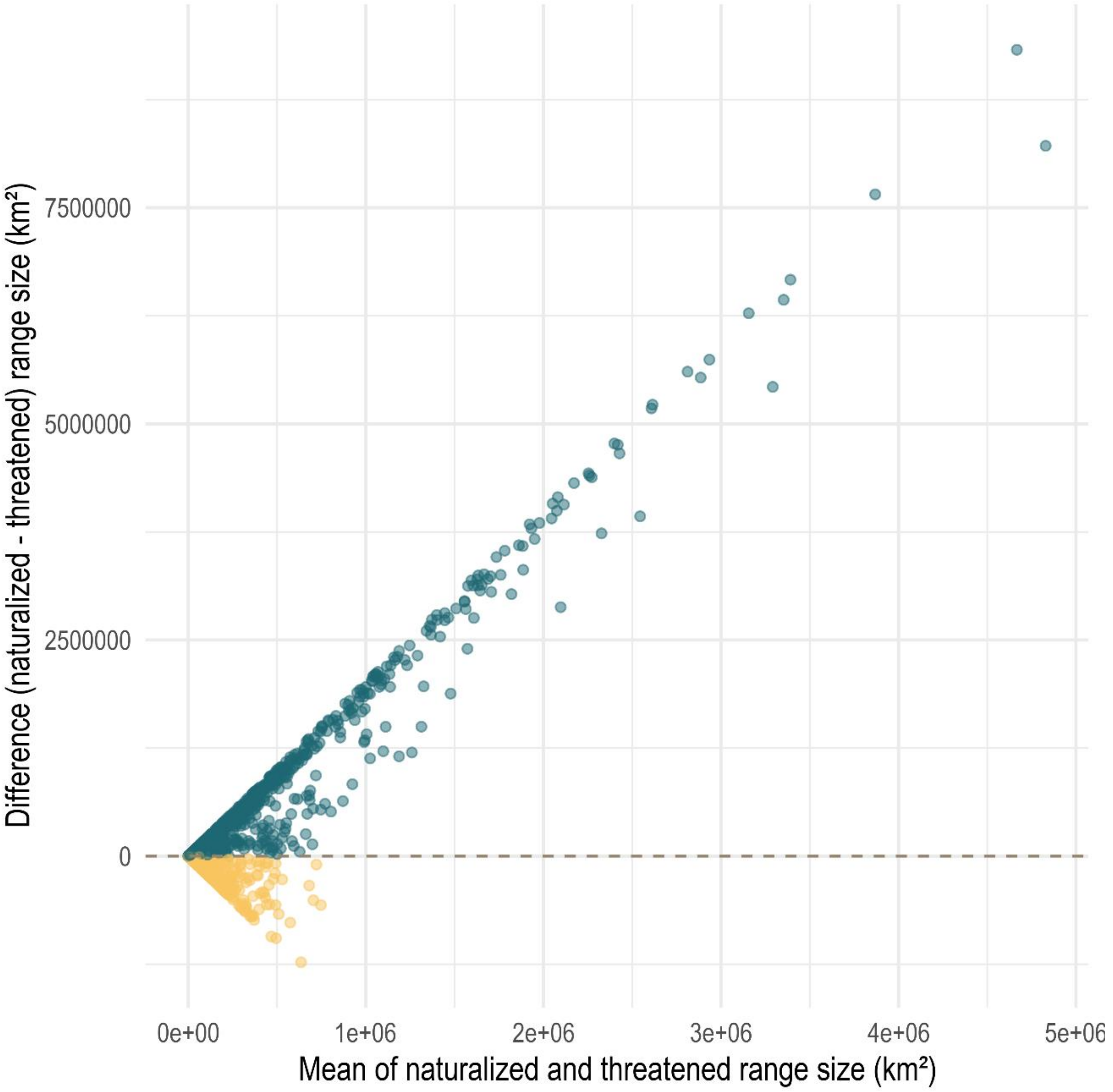

Fig. S2: **Bland–Altman plot comparing species' naturalized and threatened range size.** The plot shows the difference between the range size (measured as area of occupancy; AOO) for naturalized and threatened ranges (y-axis) against the mean AOO for both categories (i.e., [naturalized AOO + threatened AOO]/2; x-axis) for each naturalized species that is considered threatened in at least parts of its native range (n = 1,716). Each point represents a species. Green indicates a larger naturalized than threatened range, yellow the opposite. The dashed horizontal line at zero represents species with equal naturalized and threatened AOOs. AOOs may be close to zero, despite a mapping resolution of ~6,000 km² at the equator, due to distortions in area representation at higher latitudes and the cropping of native or non-native range polygons (see Methods S3).

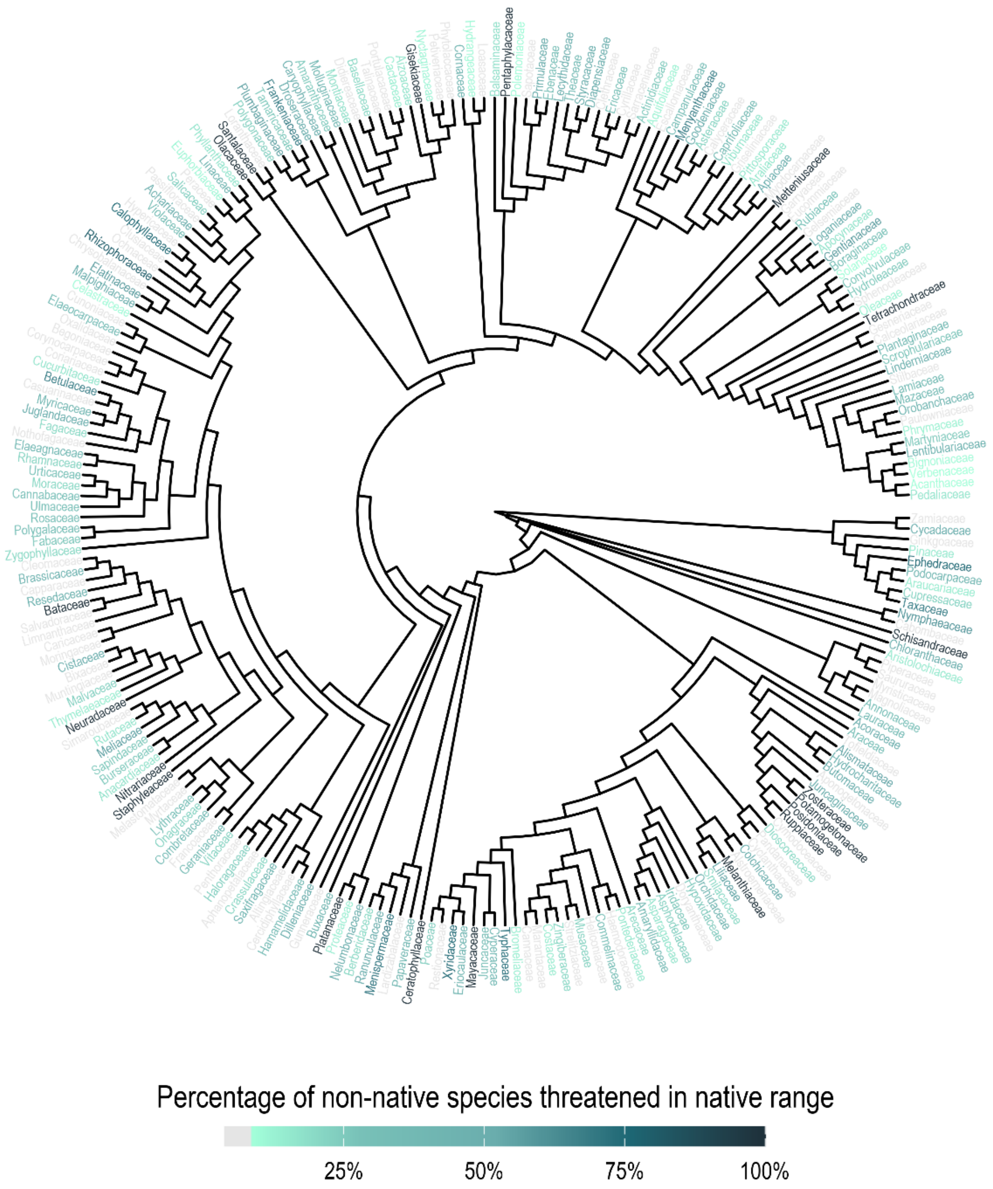

Fig. S3: **Phylogenetic distribution of plant species that are naturalized and threatened.** Phylogenetic tree of seed plant families with naturalized species, shaded to show the percentage of naturalized species considered threatened in part of their native ranges (Methods S4). Families shown in grey have naturalized species, but none classified as threatened in part of their native ranges.

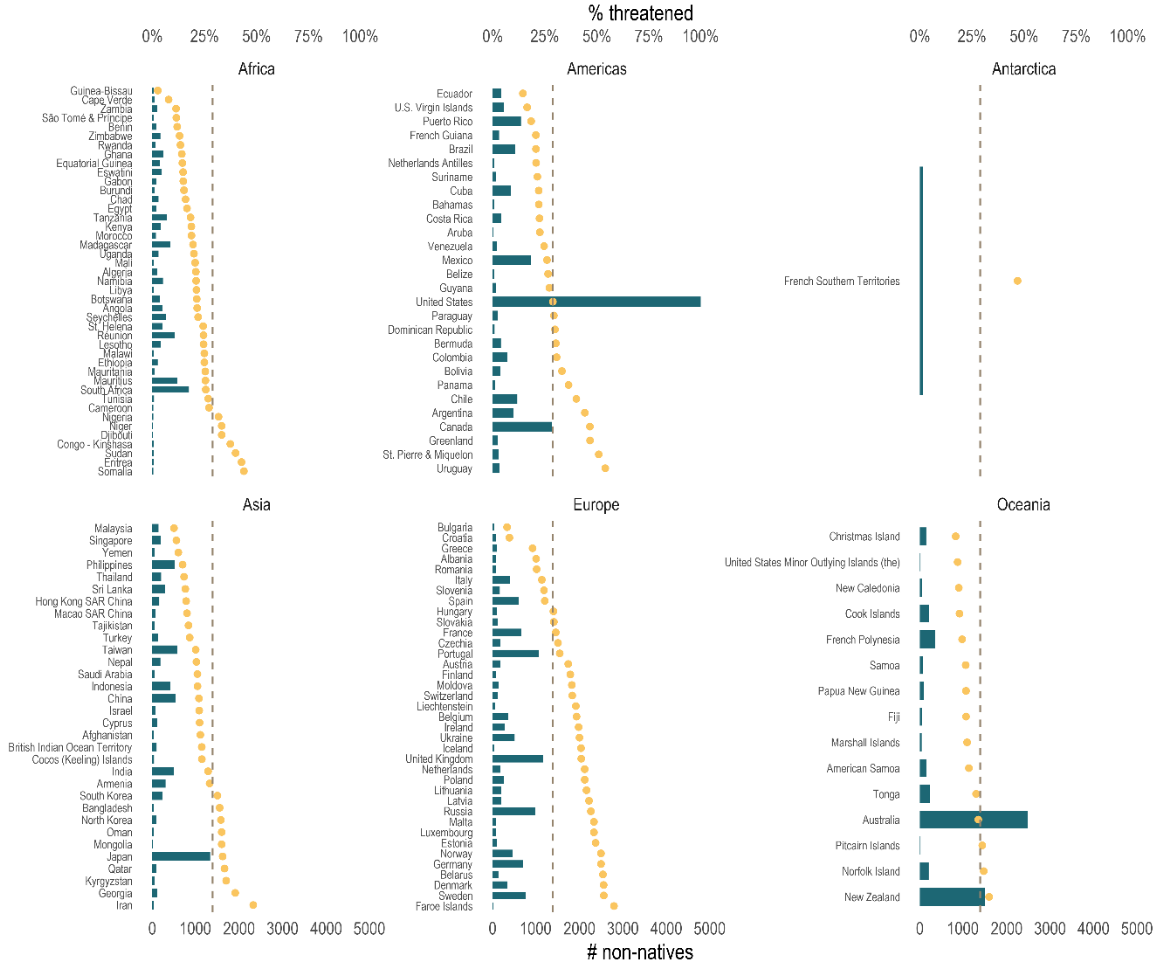

Fig. S4: **Percentage of naturalized plant species per country that are threatened elsewhere.** The number of naturalized species (green bars, lower x-axis) and the percentage of them threatened elsewhere (yellow dots, upper x-axis). The percentage was calculated by dividing the number of a country's naturalized species that are threatened in at least one country (based on our subglobal Red List synthesis) by the total number of naturalized species in that country. Only countries with more than 20 naturalized species are included (155 out of 176 countries). For example, Western Sahara and the Virgin Islands (British), in which 100% of naturalized species were part-of-native-range-threatened, were excluded from this figure due to having only one and three naturalized species recorded in GloNAF, respectively. Dashed line indicates the global average percentage of a country's naturalized flora that is partially threatened in their native ranges (29%). Comparisons between countries must be interpreted with caution, as data quality is variable (Methods S1).

References

Brown MJ, Walker BE, Black N, Govaerts RH, Ondo I, Turner R, Nic Lughadha E. **2023**. rWCVP: a companion R package for the World Checklist of Vascular Plants. *New Phytologist*.

Chamberlain S, Oldoni D, Waller J. **2022**. rgbif: interface to the global biodiversity information facility API.

Essl F, Bacher S, Genovesi P, Hulme PE, Jeschke JM, Katsanevakis S, Kowarik I, Kühn I, Pyšek P, Rabitsch W, *et al.* 2018. Which Taxa Are Alien? Criteria, Applications, and Uncertainties. *BioScience* **68**: 496–509.

Govaerts R, Nic Lughadha E, Black N, Turner R, Paton A. **2021**. The World Checklist of Vascular Plants, a continuously updated resource for exploring global plant diversity. *Scientific data* **8**: 215.

Grenié M, Berti E, Carvajal-Quintero J, Dädlow GML, Sagouis A, Winter M. **2023**. Harmonizing taxon names in biodiversity data: A review of tools, databases and best practices. *Methods in Ecology and Evolution* **14**: 12–25.

Hojsgaard D, Klatt S, Baier R, Carman JG, Hörandl E. **2014**. Taxonomy and Biogeography of Apomixis in Angiosperms and Associated Biodiversity Characteristics. *Critical Reviews in Plant Sciences* **33**: 414–427.

Holz H, Segar J, Valdez J, Staude IR. **2022**. Assessing extinction risk across the geographic ranges of plant species in Europe. *Plants, People, Planet* **4**: 303–311.

IUCN. **2012**. *Guidelines for Application of IUCN Red List Criteria at Regional and National Levels: Version 4.0*. Gland, Switzerland and Cambridge, UK: IUCN, iii + 41pp.

IUCN. **2025**. The IUCN Red List of Threatened Species. https://www.iucnredlist.org. Version 2024-2. Accessed on 25.02.2025.

van Kleunen M, Pyšek P, Dawson W, Essl F, Kreft H, Pergl J, Weigelt P, Stein A, Dullinger S, König C, *et al.* 2019. The Global Naturalized Alien Flora (GloNAF) database. *Ecology* **100**: e02542.

Li H-T, Luo Y, Gan L, Ma P-F, Gao L-M, Yang J-B, Cai J, Gitzendanner MA, Fritsch PW, Zhang T, *et al.* 2021. Plastid phylogenomic insights into relationships of all flowering plant families. *BMC Biology* **19**: 232.

Massicotte P, South A, Hufkens K. **2023**. rnaturalearth: World map data from natural earth. *R package version 0.3* **2**.

Orme D, Freckleton R, Thomas G, Petzoldt T, Fritz S, Isaac N, Pearse W. **2013**. The caper package: comparative analysis of phylogenetics and evolution in R. *R package version* **5**: 1–36.

Pebesma EJ. **2018**. Simple features for R: standardized support for spatial vector data. *R J.* **10**: 439.

Revell LJ. **2012**. phytools: an R package for phylogenetic comparative biology (and other things). *Methods in ecology and evolution*: 217–223.

Schippmann U. **2020**. *Bibliography of national Red Lists for vascular plants*. Bundesamt für Naturschutz.

Schrader J, Weigelt P, Cai L, Westoby M, Fernández-Palacios JM, Cabezas FJ, Plunkett GM, Ranker TA, Triantis KA, Trigas P. **2024**. Islands are key for protecting the world's plant endemism. *Nature*: 1–7.

Wood S, Wood MS. **2015**. Package 'mgcv'. *R package version* **1**: 729.

Xue J, Shcherbakov AV, Kipriyanova LM, Zhu L, Ma K. **2023**. Mapping Asia Plants: The Threat Status and Influencing Factors of Rare and Endangered Vascular Plant Species in North Asia (Asian Russia). *Plants* **12**: 2792.

ZSL and IUCN National Red List Working Group. **2022**. National Red List Database. https://www.archive.nationalredlist.org. Accessed on 2024-01-05.
